# The Characteristics of White Matter Hyperintensities in Patients With Migraine

**DOI:** 10.3389/fpain.2022.852916

**Published:** 2022-06-20

**Authors:** Catherine D. Chong, Todd J. Schwedt, Meesha Trivedi, Brian W. Chong

**Affiliations:** Mayo Clinic, Phoenix, AZ, United States

**Keywords:** magnetic resonance imaging, migraine, white matter hyperintensities, FLAIR, structural imaging

## Abstract

**Background:**

The presence of white matter hyperintensities (WMHs) in migraine is well-documented, but the location of WMH in patients with migraine is insufficiently researched. This study assessed WMH in patients with migraine using a modified version of the Scheltens visual rating scale, a semiquantitative scale for categorizing WMH in periventricular, lobar, basal ganglia, and infratentorial regions.

**Methods:**

In total, 263 patients with migraine (31 men and232 women) enrolled in the American Registry for Migraine Research (ARMR) from Mayo Clinic Arizona and who had clinical brain magnetic resonance imaging (MRI) were included in this study. Those with imaging evidence for gross anatomical abnormalities other than WMHs were excluded. A board-certified neuroradiologist identified WMHs on axial T2 and fluid-attenuated inversion recovery (FLAIR) sequences. WMHs were characterized *via* manual inspection and categorized according to the scale's criteria.

**Results:**

Results showed that 95 patients (36.1%, mean age: 41.8 years) had no WMHs on axial T2 and FLAIR imaging and 168 patients (63.9%, mean age: 51.4 year) had WMHs. Of those with WMHs, 94.1% (*n* = 158) had lobar hyperintensities (frontal: 148/158, 93.7%; parietal: 57/158, 36.1%; temporal: 35/158, 22.1%; and occipital: 9/158, 5.7%), 13/168, 7.7% had basal ganglia WMHs, 49/168, 29.1% had periventricular WMHs, and 17/168, 10.1% had infratentorial WMHs. In addition, 101/168 patients (60.1%) had bilateral WMHs and 67/168 (39.9%) had unilateral WMHs (34 right hemisphere/33 left hemisphere).

**Discussion:**

Among ARMR participants who were enrolled by Mayo Clinic Arizona and who had clinical brain MRIs, nearly two-thirds had WMHs. The WMHs were the most common in the frontal lobes. Describing the features of WMHs in those with migraine, and comparing them with WMHs attributable to other etiologies, might be useful for developing classifiers that differentiate between migraine-specific WMH and other causes of WMH.

## Introduction

White matter hyperintensities (WMHs) are a common finding in magnetic resonance imaging (MRI) in people with migraine using T2-weighted and fluid-attenuated inversion recovery (FLAIR) sequences (1–3). A number of studies indicate that individuals with migraine have a higher likelihood of having WMHs (4) and there is evidence that women with migraine have a higher incidence of deep WMHs and show more rapid progression of WMHs relative to non-migraine controls (5). Several studies have investigated correlations between WMH and (1) migraine subtypes (migraine with aura vs. migraine without aura), (2) headache frequency, and (3) medication use (4, 6–8). However, conflicting study results have made it difficult to interpret whether WMHs have clinical significance in migraine (1, 3, 4, 7, 9–12). Discrepancies between studies may be due in part to variations in cohort selection, use of different MRI magnet strengths, and differences in techniques used for identifying WMHs, as some studies have used semi-automated calculation of WMHs (3, 5), whereas others have identified WMHs manually *via* routine visual identification or categorization (1, 7, 9, 10, 12). Although the significance of WMHs in migraine continues to be a matter of debate with some studies indicating no differences in the prevalence of WMHs between individuals with migraine and healthy controls this study intended to investigate the presence and characteristics of WMHs in a large migraine cohort. Furthermore, there is a lack of investigations into how WMHs attributable to migraine might be distinguishable from those attributed to other etiologies, such as small vessel ischemic disease and demyelinating disease.

**Figure 1:**
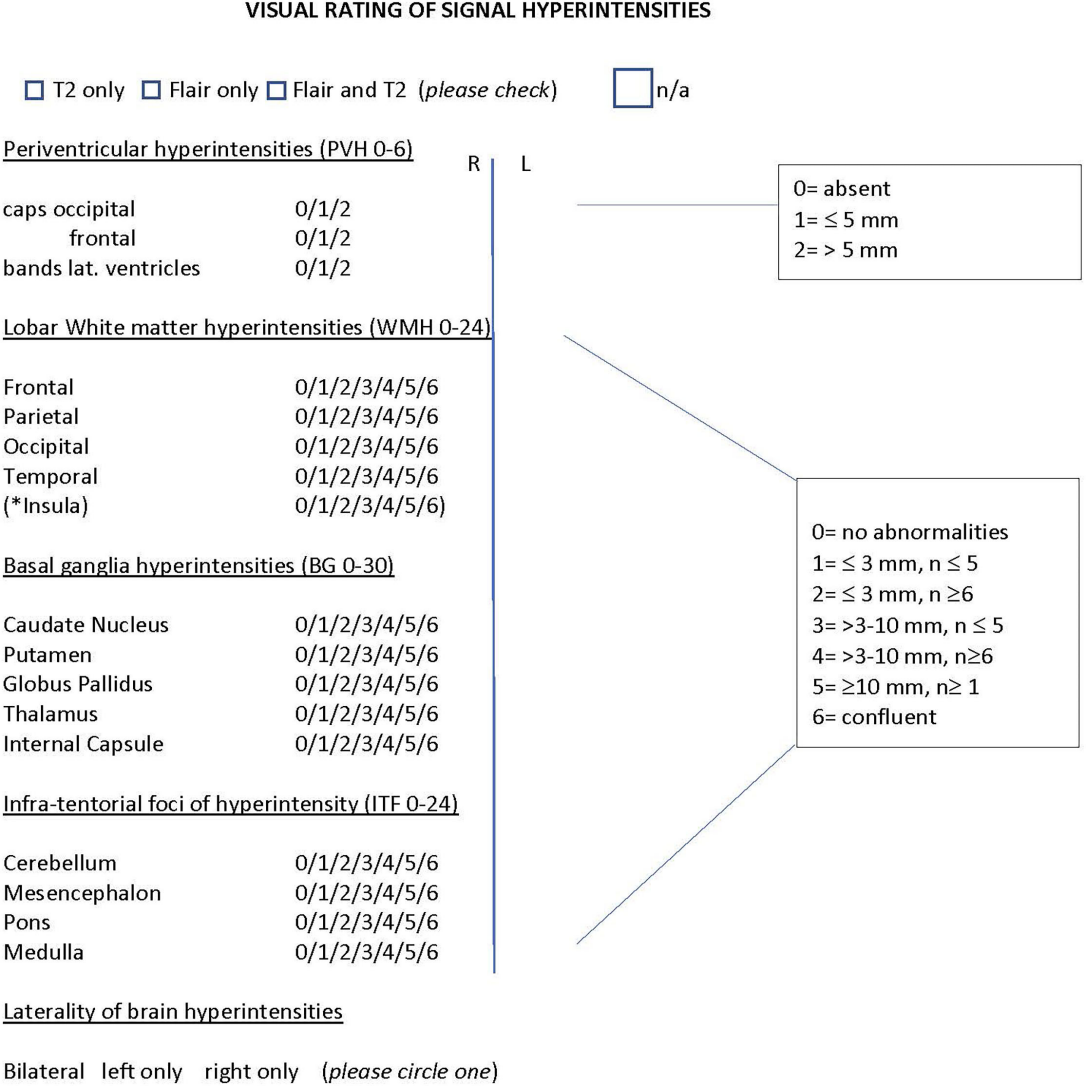
Modified Scheltens rating scale. Semiquantitative rating of signal hyperintensities for the following regions: Periventricular, lobar white matter hyperintensities (frontal, parietal, occipital, temporal), basal ganglia and infra-tentorial foci. * insular WMH were counted separately and were not included in the total of lobar WMH to remain consistent with the original rating of the scale. The number ranges in brackets indicate the range of the scale for each region. *n*= number of lesions; n/a =no abnormalities noted on scan. The scaling criteria for the regions is shown on the right side. Only those patients that had both Flair and T2 imaging were included in the analysis.

The goal of this study was to assess WMHs in patients with migraine using a modified version of the Scheltens visual rating scale (13), a well-known semi-quantitative rating scale for assessing WMHs in the following brain regions: periventricular, lobar, basal ganglia, and infratentorial (as shown in Figure 1). This study aimed to categorize WMHs in patients with migraine by their size and location, so as to allow *for future* differentiation of migraine-specific WMHs from those attributable to other diseases, such as small vessel ischemic disease and multiple sclerosis.

## Methods

### Subject Eligibility and Consent

Institutional Review Board approval was received from Mayo Clinic and all subjects completed signed consent prior to the start of this study. All migraine subjects between the ages of 18 and 80 years who were enrolled in the American Registry for Migraine Research (ARMR) from Mayo Clinic Arizona and who had brain magnetic resonance imaging (MRI) as part of their clinical care were selected for this study. ARMR methodology has been previously published (14). Subjects were included in this study if both FLAIR and T2 imaging sequences were available. Those patients that had only FLAIR or only T2 sequences were excluded from the analysis. Subjects were excluded if imaging reports identified brain abnormalities other than WMHs and if there were significant imaging artifacts due to motion, oral cavity fillings, or other etiologies.

**Figure 2:**
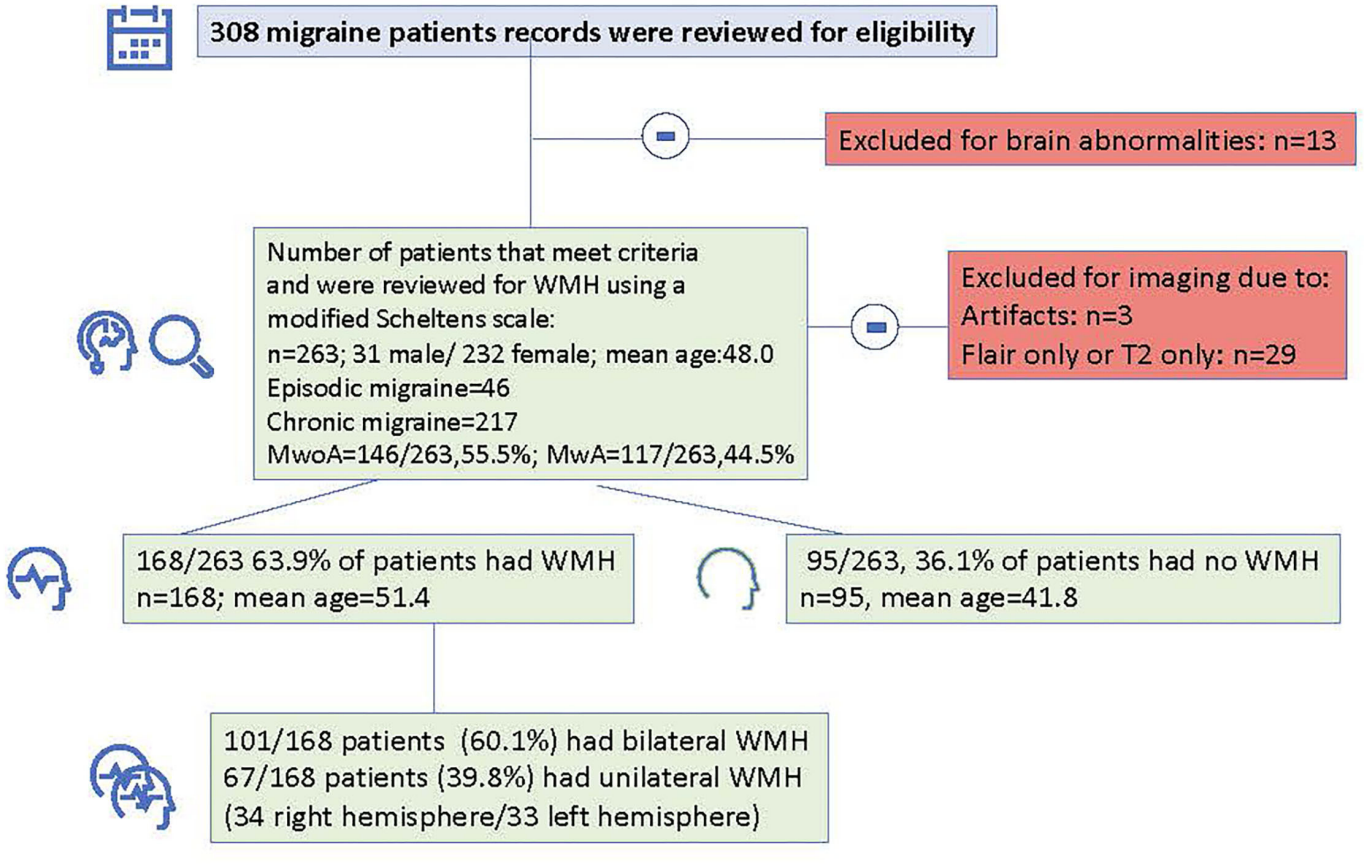
Flowchart indicating total number of subjects that were reviewed, number of patients that were excluded and number and demographic information for patients with and without white matter hyperintensities.

Figure 2 shows a flowchart of the study design. Medical records were reviewed for 308 subjects. Thirteen subjects who had abnormal brain findings were excluded. Following imaging review, three patients were excluded for imaging artifacts, and twenty-nine patients were excluded who only had usable T2 or only had FLAIR imaging, thus leaving a total of 263 migraine subjects with good quality T2 and FLAIR imaging.

### Brain MRI Evaluation

All images were read by a single, board-certified neuroradiologist (BC) with 30 years of experience. As part of this study, to keep the radiologist uninformed of each patient's diagnosis, the neuroradiologist was also given imaging of those with cluster headache and post-traumatic headache with and without WMHs. However, data from patients with cluster headache and post-traumatic headache, were not included in the analyses reported herein. All Images were read over a 4-month period (September 2020–January 2021) using the desktop viewer QREADS (9), which was integrated with the picture archiving and communication system (PACS) (10). All imaging was conducted at Mayo Clinic, Arizona.

White matter hyperintensities were identified as hyperintense on both FLAIR and T2 images relative to the surrounding brain parenchyma. The widest dimension of the lesion was measured with digital calipers and the location was documented using a modified version of the Scheltens visual rating scale (13) (Figure 1). The scale was modified to identify scanning sequences (those patients who only had T2 or only had FLAIR imaging were excluded) and to account for laterality of WMHs (bilateral or unilateral), image quality, and the presence of insula WMH, although these were counted separately and were not included in the total scoring of lobar WMH, to not alter the scoring criteria of the original scale.

The Scheltens scale allows scoring of WMHs by relative size and number of lesions. Periventricular hyperintensities are calculated for three main locations: occipital caps, frontal caps, and lateral bands. Each of these is scored as either, 0 (absent), 1 (≤ 5 mm), or 2 (>5 mm). The total score for periventricular hyperintensities ranges from 0 to a maximum total of 6.

Lobar, basal ganglia, and infratentorial hyperintensities are scored using the following scaling criteria: 0 (absent), 1 (≤3 mm and the number of lesions ≤ 5), 2(≤ 3 mm and the number of lesions ≥6), 3 (>3 mm and the number of lesions ≤ 5), 4 (>3 mm and the number of lesions ≥6), 5 (≥10 mm and the number of lesions ≥1), and 6 (lesion formations are confluent). Lobar WMHs are calculated for frontal, parietal, occipital, and temporal regions. The total score for lobar WMHs ranges from 0 to a maximum score of 24. Total scores for basal ganglia regions (caudate nucleus, putamen, globus pallidus, thalamus, and internal capsule) range from 0 to a maximum score of 30. Infra-tentorial foci of hyperintensity are calculated for the cerebellum, mesencephalon, pons, and medulla. The total score ranges from 0 to a maximum score of 24.

### Statistical Interpretation

Subject demographics and headache characteristics and data that were recorded from the modified Scheltens Visual Rating scale were organized in Excel and exported to SPSS 26 (Released 2019. IBM SPSS Statistics for Macintosh, Version 26.0. Armonk, NY: IBM Corp) for statistical interpretation. Demographic information and data from the Scheltens visual rating scale were compared using two-tailed *t*-tests or Fisher's exact tests, or chi-square tests as appropriate. A binary logistic regression analysis was used for a *post-doc* analysis to explore the relationship between headache frequency and WMHs.

## Results

The 263 migraine patients (age range: 18–80 years) had an average age of 48.0 years (SD = 15.0), and 88.2% were women. The average headache frequency reported within the electronic medical record was 18.7 (SD = 10.9) days per month. Forty-six patients had episodic migraine and 217 had chronic migraine. In addition, 117/263 patients (44.5%) had migraine with aura.

### Classification According to a Modified Version of the Scheltens Scale

On axial T2 and FLAIR imaging, 95/263 patients (36.1%: mean age: 41.8 years and SD = 13.4) had no WMHs, while 168/263 patients (63.9%, mean age: 51.4 years and SD = 14.7) had WMHs. WMHs were most common in lobar regions, 94.1% (*n* = 158) had lobar hyperintensities (frontal: 148/158, 93.7%; parietal: 57/158, 36.1%; temporal: 35/158, 22.1%; and occipital: 9/158, 5.7%).

In addition, Thirteen patients (7.7%) had basal ganglia WMHs, 49 (29.1%) had periventricular WMHs, and 17 (10.1%) had infratentorial WMHs.

For patients with frontal lobe lesions, 87 patients (58.8%) had lesions of 3 mm or less in size and 61 patients (41.2%) had lesions over 3 mm in size (*p* =.0.09). Additionally, 36 patients (63.2%) had WMHs in the parietal lobe of 3 mm or less in size and 21 patients (36.8%) had WMHs over 3 mm in size (*p* = 0.009).

For patients with temporal lesions, 24 patients (68.6%) had WMHs of 3 mm or less in size and 11 patients (31.4%) had WMHs over 3 mm in size (*p* = 0.001). Four patients had occipital WMHs of 3 mm or less in size. There were no patients who had occipital WMHs that were over 3 mm in size.

Of 148 patients, 21 patients (14.2%) with frontal lobe WMHs had WMHs in the insula. Of those, the majority of WMHs of 3 mm or less in size [17 patients (81%) had insular WMHs of 3 mm or less in size and 4 patients had insular WMH larger than 3 mm (19%); *p* = 0.001].

**Figure 3:**
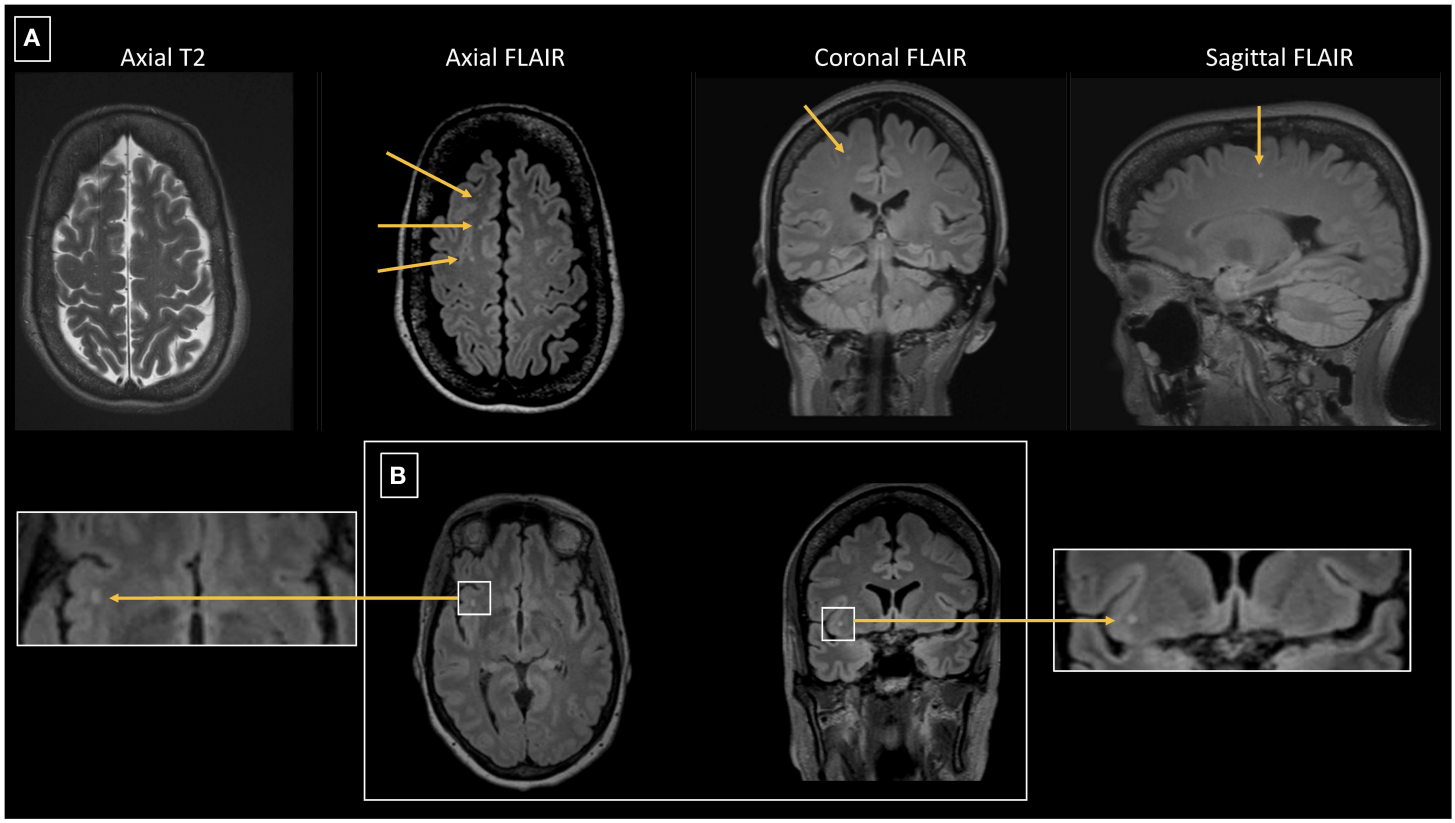
**(A)** 64 year-old female with episodic migraine with aura who averaged 5 migraine attacks per month. No history of vascular risk factors. Axial FLAIR images show 3 of 9 right frontal white matter lesions all of which are small and punctate in contour. Lesions are less conspicuous on axial T2 images than seen on FLAIR images. One of the lesions is depicted on coronal and sagittal FLAIR images. **(B)** Axial and coronal FLAIR images in the same patient show a punctate lesion in the right insular white matter.

**Figure 4:**
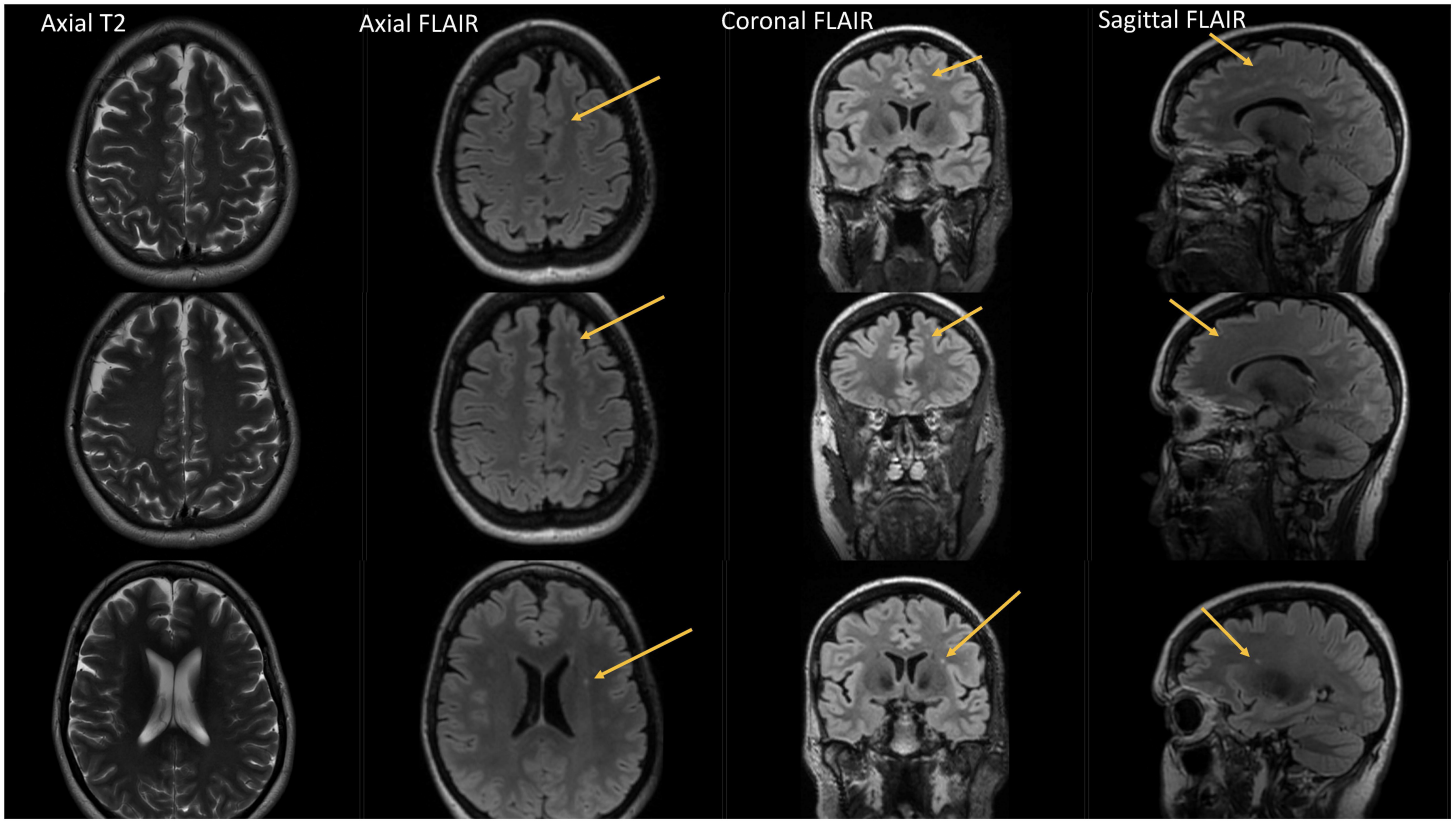
Fourty-eight year-old female with chronic migraine, no aura. No history of vascular risk factors. Axial, coronal and sagittal FLAIR images show 3 left frontal white matter lesions all of which are small and punctate in contour. Lesions are difficult to appreciate on axial T2 images.

Furthermore, 101/168 patients (60.1%) had bilateral WMHs and 67/168 patients (39.9%) had unilateral WMHs (34 right hemisphere/33 left hemisphere). Figures 3, 4 show representative migraine subjects with frontal WMHs of typical size and distribution seen in the studied cohort. Table 1 shows lobar (frontal, temporal, parietal, and occipital) WMHs and periventricular (frontal, occipital, and lateral bands) WMHs categorized by lesion size. These descriptive data indicate that patients with larger WMHs tended to be older. Fewer than 2% of patients who did not have WMHs in the frontal lobe had WMHs in the basal ganglia or infratentorial regions. About 30.0% of patients with WMHs did not have WMHs reported in their clinical radiology reports. When comparing individuals with WMHs to individuals without WMHs, the following was found: those with WMHs were significantly older than those without WMHs (individuals with WMHs: mean age = 51.4 years, SD = 14.7; individuals without WMHs: mean age = 41.8 years, SD = 13.4; *p* < 0.001). Individuals with WMHs had significantly more years with a headache than those without WMHs (individuals with WMHs: mean years lived with headache = 21.3 years, SD = 17.6; individuals without WMHs: mean years lived with headache = 14.3 years, SD = 12.0; *p* < 0.001). There was no difference in sex (individuals with WMHs: 19 men and 149 women; individuals without WMHs: 12 men and 83 women; *p* = 0.84) and there was not a difference in episodic vs. chronic migraine (individuals with WMHs: 28 had episodic migraine and 140 had chronic migraine; individuals without WMHs: 18 had episodic migraine and 77 had chronic migraine; *p* = 0.73) or difference in aura status (individuals with WMHs: 74 had migraine with aura and 94 had migraine without aura; individuals without WMHs: 43 had migraine with aura and 52 had migraine without aura; *p* = 0.89) between individuals with WMHs compared with those individuals without WMHs. There was no difference between migraine patients with and without WMHs for the following vascular comorbidities: body mass index, diabetes mellitus or prediabetes, dyslipidemia, hypertension, cardiovascular disease, and cerebrovascular disease. There were significant group differences in smoking. About 7.1% of migraine patients without WMHs were current smokers compared with 1% of migraine patients with WMH (*p* = 0.004), as shown in Table 2. An exploratory binary logistic regression analysis indicated that there was no association between headache frequency (i.e., episodic vs. chronic migraine) and WMHs (odds ratio (*OR*).756; 95% *CI*.339–1.686; *p* = 0.494). In the opinion of the neuroradiologist who reviewed the imaging, WMHs tended to be round (punctate) in shape and not confluent.

**Table 1:** Shows the mean ages of migraine patients with WMH for specific locations categorized by size. n/a = no WMH for that size and location.

**Periventricular** **WMH per location**	**≤ 5 mm**	**> 5 mm**
caps occipital mean age (SD)	53.5 (16.7)	62.1 (11.2)
caps frontal mean age (SD)	51.5 (14.7)	62.5 (12.2)
bands mean age (SD)	n/a	64.0 (10.9)
**WMH per Lobar** **location**	**≤ 3 mm**	**>3 mm**
Frontal mean age (SD)	49.05 (13.5)	57.8 (14.0)
Parietal mean age (SD)	55.1 (15.6)	60.0 (10.2)
Occipital mean age (SD)	63.4 (8.9)	n/a
Temporal mean age (SD)	61.67 (12.2)	56.5 (11.7)
Insula Mean age (SD)	54.1 (14.2)	57.25 (9.5)

**Table 2:** Demographic information and vascular comorbidities of individuals with and without white matter hyperintensities.

	**WMH *yes*** **(*n* = 168)**	**WMH *no*** **(*n* = 95)**	**p-value**
***Demographics***			
Age Mean (SD)	51.4 (14.7)	41.8 (13.4)	<0.001
Sex male/female	19/149 (11.3%/88.7%)	12/83 (12.6%/87.4%)	0.84
Episodic/chronic migraine n (%)	28/140 (16.7%/83.3%)	18/77 (18.9%/81.1%)	0.73
Aura yes/no	74/94 (44.0%/56.0%)	43/52 (45.3%/54.7%)	0.89
Years lived with headache Mean (SD)	21.3 (17.6)	14.3 (12.0)	<0.001
***Vascular comorbidities***			
BMI Mean (SD) *normal n* (%) *overweight n* (%) *obese n* (%)	29.5 (15.0) 64 (38.1%) 40 (23.8%) 64 (38.1%)	28.7 (8.9) 37 (38.9%) 23 (24.2%) 35 (36.8%)	0.64 0.89 0.99 0.89
Diabetes mellitus *or* prediabetes *n* (%)	18 (10.7%)	9 (9.5%)	0.83
Smoking/Tobacco use			
*current smokers n* (%)	1 (0.6%)	7 (7.4%)	0.004
*past smokers n* (%)	51 (30.4%)	18 (18.9%)	0.057
*no smoking history* •*n* (%)	116 (69%)	70 (73%)	0.48
Dyslipidemia *n* (%)	52 (31%)	23 (24.2%)	0.26
Hypertension *n* (%)	35 (20.8%)	24 (25.3%)	0.44
Cardiovascular disease *n* (%)	11 (6.5%)	5 (5.3%)	0.79
Cerebrovascular disease *n* (%)	10 (6%)	4 (4.2%)	0.77

*BMI, body mass index; WMH, white matter hyperintensities; SD, standard deviation; Years lived with headache, number of years lived with Headache.*

*Statistical significance was tested using independent T-test or Fisher's Exact Test, as appropriate*.

## Discussion

White matter hyperintensities classification based on a modified version of the Scheltens visual rating scale indicated that a majority of patients with migraine (63.9%), all of whom were enrolled in a headache specialty clinic and had brain MRI as part of their clinical evaluation, had WMHs on T2 and FLAIR imaging, which were most prominent in the frontal lobes. Fewer than 2% of patients who did not have WMHs in the frontal lobe had WMHs in the basal ganglia or infratentorial regions and only 5.6% had WMHs in the periventricular WM. WMHs were mostly distributed bilaterally (61%) and lesions in the right and left hemisphere occurred with equal frequency.

Our results are in accordance with those from Xiu and colleagues who assessed WMHs in 69 patients with migraine, 24 of whom had WMHs. Similar to our results, Xiu reported WMHs to be most prevalent in the frontal lobe (74.9%) followed by the parietal lobes. In our study, 14% of individuals (*n* = 21) with frontal lobe hyperintensities had WMHs located in the insula which is an intriguing finding as the insula is a known ‘cortical hub' and a key region of the salience network (15) involved in cognitive and interoceptive components of the pain experience (16) and an area known in migraine to demonstrate alterations brain functional connectivity using resting-state imaging (17–19). Furthermore, using positron emission tomography (PET) [^11^ C]HOwe (8) PBR28 imaging, Hadjikhani and colleagues found increased binding of the 18 kDa translocator protein, a marker of glial activation, in individuals with migraine with aura in the bilateral insula (20). Furthermore, the authors also noted a positive correlation between the insula [^11^ C] PBR28 standard uptake ratio and migraine attack frequency, further suggesting insula involvement in neuroinflammation and nociception.

In the present study, patients with migraine who had WMHs were significantly older compared to patients with migraine who did not have WMHs. Furthermore, those who were older tended to have larger WMHs for lobar (frontal, temporal, parietal, and occipital) and periventricular regions compared with younger patients. Frazekas reported that 11% of symptom-free subjects (*n* = 87, ages 31–83 years) had WMHs in the 4th decade of life which increased to 83% in subjects over the age of 70 years. This percentage was even higher in those with cardiovascular risk factors (21). It is possible that the larger WMHs found in our study could be at least partially attributable to factors other than migraine.

There is a continuous debate about whether WMHs have clinical significance in migraine and more research is needed that defines the characteristics of WMH attributed to migraine (i.e., size, shape, number, and distribution) and differentiates them from those associated with other diseases, such as small vessel ischemic disease, lacunar infarcts, and multiple sclerosis. In our study, the majority of lobar WMHs in patients with migraine were smaller than 3 mm in diameter. In the opinion of the neuroradiologist who reviewed the imaging, WMHs were most commonly punctate in shape. None of them were visible on T1-weighted imaging as hypointense, as can be seen with lacunar infarcts, small vessel ischemic white matter changes, and multiple sclerosis. Furthermore, none of the lobar lesions in our migraine cohort were confluent (i.e., none had a score of 6 on the Scheltens scale), as can be seen in these other three entities. An additional observation in our migraine cohort was that few patients had periventricular WM lesions contiguous with the lateral ventricles or capping of the lateral ventricles. Similarly, few patients had involvement of the basal ganglia, brainstem, or other posterior fossa structures. These features might prove unique to migraine and be useful for differentiating WMHs attributable to migraine from those due to other diseases. Interestingly, 30% of those with WMHs identified using the Scheltens scale did not have WMHs identified in the clinical imaging report. Possible explanations for a negative clinical report in 30% of the cases could be that either the lesions were too small to be appreciated, or WMHs were not felt to be clinically relevant and therefore not described. As there is a paucity of studies that have used systematic methods to assess and compare WMHs between disorders, at times it can be difficult in clinical practice to determine if WMHs are due to migraine or if they are better explained by another etiology (22). Classification of WMHs using the modified Scheltens scale may contribute to better identification and understanding of the distribution of migraine WMHs, which could help to clarify the relationship between WMHs and headache features.

The distribution and size of WMHs in our migraine cohort seem to differ from that of focal WMHs associated with lacunar infarcts. Ryu et al. (23) used a modified version of the Scheltens scale to assess lacunar infarcts and found a left-hemisphere dominance of WMHs in the corona radiata, basal ganglia, thalamus, and internal capsule. Lesions seen in lacunar infarcts are usually small lesions that are hyperintense on T2 and FLAIR and have low signal on T1, correlating with pathological descriptions of gliosis and encephalomalacia, respectively (24). The absence of a low signal on T1 in this migraine cohort might imply that these lesions are not due to encephalomalacia. Similar to lacunar infarcts, multiple sclerosis lesions are bright on T2 and FLAIR images and can demonstrate a low T1 signal when the lesions are chronic (25). The absence of a low T1 signal in lesions seen in our migraine cohort distinguishes the WMHs lesions from lacunar infarcts and multiple sclerosis. It is interesting that even in older patients with migraine, WMH are not low signal and therefore may not represent a final common pathway of encephalomalacia that is seen with lacunar infarcts and demyelination.

The results of this study and future investigations could have meaningful implications for the management of patients who have migraine. Whether indicated or not, many patients with migraine undergo brain MRI, either due to the presence of “red flag” features that increase the likelihood of a secondary headache or simply due to clinician and patient anxiety about missing an underlying diagnosis (26, 27). Many of these brain MRIs will demonstrate WMHs. At times, it is relatively straightforward to assign an etiology to these WMHs, such as attributing a couple of frontal, small, punctate WMHs to migraine in a young patient with no vascular risk factors and no history of symptoms to suggest demyelinating disease or stroke. However, the situation is often not straightforward, such as when WMHs are identified in an older patient with migraine and several vascular risk factors. In this situation, for example, it can be unclear if the WMHs are attributable to migraine or small vessel ischemic disease. Identifying characteristic features of WMH attributable to migraine and developing classification models for WMHs that are likely attributable to migraine could assist the clinician in determining if there is a need for additional diagnostic testing, such as might be the case if the WMHs cannot be attributed to migraine. Future studies will compare WMH features among those with migraine and other diseases that are associated with WMHs, to develop such classification models.

### Limitations

There are several limitations to our study. All subjects included in this study had brain MRIs ordered for clinical reasons. Thus, they might have specific characteristics that led the clinician to order an MRI (e.g., atypical symptoms and abnormal neurological examinations). Although the Scheltens scale has adequate intra- and inter-observer reliability (13), we acknowledge that having only one neuroradiologist review, the scans could have affected the validity of the study findings, despite the fact that the neuroradiologist was kept uninformed of each patient's diagnosis.

The reliability and validity of our study findings will need to be assessed using future studies that include larger patient cohorts and multiple readers. Such prospectively designed studies, which will enroll younger cohorts of patients with episodic and chronic migraine and exclude subjects with vascular risk factors are needed to verify our current findings. All subjects in this study were enrolled in a headache specialty clinic, and the results might not be generalizable to the general population of people with migraine. It is possible that some of the enrolled individuals had additional vascular risk factors which were not assessed as part of this study or other conditions which might increase their risk of having WMHs attributable to conditions other than migraine. Lastly, as the age range of this study cohort was broad, it cannot be ruled out that some of the WMHs found in older subjects were age-related and not necessarily related to migraine.

## Conclusion

White matter hyperintensities among those with migraine were most common in the lobar regions and were more commonly under 3 mm in diameter. Periventricular WMHs were uncommon, as were basal ganglia and infratentorial WMHs, especially in the absence of frontal WMHs. These and other characteristics might help the clinician to differentiate WMHs attributed to migraine from those attributed to other diseases. Furthermore, WMHs seen in patients with migraine tended to be punctate (< 3 mm in size) and not confluent. Future investigations should directly compare and contrast WMHs associated with migraine with those associated with other diseases, to develop an easy-to-use model for the classification of WMHs attributed to migraine.

## Key Findings

- Using T2 and FLAIR imaging, WMHs were detected in the majority of patients with migraine using a modified version of Scheltens visual rating scale.
- Most of the lobar WMHs were under 3 mm in size.
- When comparing migraine individuals with and without WMHs, there was not a difference in the distribution of sex ratios (male vs. female), the presence or absence of aura, or ratios of episodic vs. chronic migraine between cohorts.

## Data Availability Statement

Researchers wishing to access our data should send their request via e-mail to the corresponding author (Chong.catherine@mayo.edu) and the Mayo Clinic Institutional Review Board (ude.oyam@EBRI).

## Ethics Statement

The studies involving human participants were reviewed and approved by Mayo Clinic IRB. The patients/participants provided their written informed consent to participate in this study.

## Author Contributions

CC, TS, MT, and BC designed the study, collected and analyzed the data, and drafted the manuscript. All authors contributed to the article and approved the submitted version.

## Conflict of Interest

The authors declare that the research was conducted in the absence of any commercial or financial relationships that could be construed as a potential conflict of interest.

## Publisher's Note

All claims expressed in this article are solely those of the authors and do not necessarily represent those of their affiliated organizations, or those of the publisher, the editors and the reviewers. Any product that may be evaluated in this article, or claim that may be made by its manufacturer, is not guaranteed or endorsed by the publisher.

## Abbreviations

BDI: Beck Depression Inventory
MRI: magnetic resonance imaging
SD: standard deviation
WMHs: white matter hyperintensities.
